# Computer-Based Development of Reading Skills to Reduce Dropout in Uncertain Times

**DOI:** 10.3390/jintelligence10040089

**Published:** 2022-10-21

**Authors:** Katalin Szili, Renáta Kiss, Benő Csapó, Gyöngyvér Molnár

**Affiliations:** 1 Institute of Education, Kaposvár Campus, Hungarian University of Agriculture and Life Sciences, 7400 Kaposvár, Hungary; 2 MTA—SZTE Research Group on the Development of Competencies, Institute of Education, University of Szeged, 6722 Szeged, Hungary; 3 MTA—SZTE Digital Learning Technologies Research Group, Institute of Education, University of Szeged, 6722 Szeged, Hungary

**Keywords:** reading, computer-based development, reading skills training program

## Abstract

An adequate level of reading comprehension is a prerequisite for successful learning. Numerous studies have shown that without a solid foundation, there can be severe difficulties in later learning and that failure in the first years of schooling can determine attitudes to learning. In the present study, we present the effect size of an online game-based training program implemented on eDia. The primary goals of the development program are to develop fluency in reading and reading comprehension in Grades 3–4. The content of the program has been developed in accordance with the national core curriculum and the textbooks based on it. Therefore, it can be integrated into both classroom-based lessons and extracurricular activities outside of class. The quasiexperimental research involved 276 students. Propensity score matching was used in examining the effect size of the development program to increase the validity of the results. Through the training program, the development of students in the intervention group accelerated greatly (d = .51), which proved to be even higher in the lowest and average skill groups (d_1_ = 1.81; d_2_ = .92) as well as in the disadvantaged student group (d = .72). Latent-change analyses confirmed the sensitivity, relevance, and importance of developing comprehension at 9–10 years of age and the generalizability of the results (χ^2^ = 421.5; df = 272; *p* < .05; CFI = .950; TLI = .945; RMSEA = .045 (CI: .036, .153). The study provided evidence that a well-designed online training program is suitable for developing comprehension and overcoming disadvantages, even without the presence of the teacher outside the classroom.

## 1. Introduction

The role of reading comprehension is indispensable in learning and in a proper understanding of instructions, and it is becoming increasingly important in modern societies. Any lack of reading can severely limit an individual’s ability to succeed (Wyschkon et al. 2017; Luo et al. 2017; Jamshidifarsani et al. 2019). A person who reads well makes a continuous effort to search for information from the text to interpret it. If efficiency is inadequate, the reader is unable to memorize information, make connections, and integrate background knowledge with what is read (National Reading Panel 2000). The integration of experience and background knowledge into what is read is an essential condition for the development of intelligent and fluent reading (Nelson et al. 2012), and the lack of this skill puts students at risk for failure in school and dropout (Rabiner et al. 2016).

Continuous improvement of students’ reading skills should continue to be a priority after the lower grades, as research identifies a decline in the reading performance of low-income students after third grade (Chall and Jacobs 1983; Hirsch 2003; Stockard 2010; Campbell et al. 2019). Teachers need continuous feedback on how their students are developing in different areas of reading to support effective reading instruction (Sztajn et al. 2012). Thanks to technology, the continuity of feedback can now be integrated into the process of modern teaching. These aspects have led researchers to take steps to design development programs that are available online, provide continuous feedback to students, and therefore offer an appropriate way to develop the most critical skills for successful reading comprehension, even without face-to-face contact.

We already know a great deal about the components of effective classroom and individual reading instruction, but more research is needed to assess the impact of reading interventions that align with and complement the technology-based curriculum. This issue has become a hot topic in uncertain times, when the lack of personal school instruction has resulted in a significant learning gap even in the most important domains of education—reading, mathematics, and the sciences—especially among students in the lower grades (Engzell et al. 2021; Tomasik et al. 2021; Molnár and Hermann 2022). This study expands our current knowledge of the potential of implementing computer-based training for reading skills beyond normal teaching hours to bridge the learning gap that has grown during remote learning due to COVID-19 at the beginning of schooling among students at the ages of 9–11. An online, curriculum-based training program in support of text comprehension will be presented, the effectiveness of which will be evaluated after the intervention and three months later. The validity of the quasiexperimental study was improved by matching propensity scores to assign students to intervention and control groups. This is the first available online training program in Hungarian, which has been empirically tested and which focuses on the development of the reading skills of students aged 9–11. Thus, the focus of this study is twofold: first, to show that the age of 9–11 is a sensitive period for accelerating the development of reading skills, and, second, to demonstrate that a computerized, personalized training program can prevent broader learning gaps in reading skills even at this stage of education with immediate, continuous, and differentiated feedback without the presence of the teacher.

### 1.1. Cognitive and Linguistic Components of Reading

Reading is one of the most complex and significant cognitive activities that a person engages in (Kendeou et al. 2016; Elleman and Oslund 2019). In the process of reading, the reader creates meaning through interaction with the text (Kim and Goetz 1995). Comprehension of a text requires coordination of several linguistic and cognitive processes (Castles et al. 2018), including word reading skills, working memory, generation of conclusions, monitoring comprehension, vocabulary, and prior knowledge (Perfetti et al. 2005). Importantly, it can be concluded that in the process of reading, the higher-level complex skills of decoding and text comprehension require integration of several basic reading and reading-related skills (Morris and Lonigan 2022). Reading comprehension is supported by both word reading skills (decoding) and oral language comprehension (Kendeou et al. 2009; Lonigan 2015). Decoding skills entail the integration of orthographic knowledge and phonological awareness. Comprehension skills involve integration of semantic and syntactic knowledge and inference processes. All of these basic reading skills are supported by general intelligence (Morris and Lonigan 2022). Reading has a causal effect on more general cognitive abilities; that is, it can improve overall intelligence (Gottfredson 1997). Therefore, the development of reading skills can also promote the development of intelligence (Torgesen 2005) in a classroom environment. Reading improves verbal intelligence (Cunningham and Stanovich 1991, 1998; Cotton and Crewther 2009; Cain and Oakhill 2011). Better reading skills can improve knowledge of specific facts, but it can also allow a person to acquire abstract thinking skills; thus, in addition to verbal abilities, it is also associated with an increase in nonverbal abilities (Ritchie et al. 2014).

Understanding what you read requires each component to work properly. If any part is damaged or stuck in its development, it will also affect the other components. In the early stages of learning reading, reading success is determined by a number of components, of which those related to language skills stand out (Lonigan et al. 2008). Therefore, it is necessary to understand reading as a language skill. Reading is the language skill for which the appropriately functioning spoken language skills of phonological (interpreting the sounds of speech), semantic (interpreting the actual meaning of sentences), syntactic (interpreting the grammatical structure of sentences), and pragmatic (interpreting the context of a text) organization are essential (Lonigan et al. 2008; Kamhi and Catts 2012). Based on a meta-analysis, the National Reading Panel (2000) concluded that a program that includes the following four areas of reading instruction is successful: (1) teaching phonemic awareness, (2) building phonics, (3) systematic improvement and development of fluency, and (4) strengthening comprehension. The panel found that a combination of these techniques makes reading instruction more effective. Ehri et al. (2001) identified five main pillars for success in reading acquisition: (1) developing phonological awareness, (2) building a thorough knowledge of letter-sound relationships, (3) developing vocabulary, (4) developing reading fluency, and (5) mastering comprehension strategies. These pillars are closely interlinked. Strong phonological awareness is the basis for building letter-sound relationships. A thorough knowledge of phonological awareness and letter–sound relationships facilitates the development of fluent reading, which enables the reader to access the meaning of written texts using vocabulary and comprehension strategies (Ehri et al. 2001; Kamhi and Catts 2012).

The goal of acquiring different reading skills, from phonemic awareness to vocabulary acquisition and fluency, is to be able to understand texts effectively (Jamshidifarsani et al. 2019). A variety of skills are involved in reading comprehension at an appropriate level, and the lack of one or more of these skills can impair comprehension (Nation 2005; Kendeou et al. 2014). Therefore, everyone with reading difficulty may have different levels of difficulty reading the text, resulting in a different reading profile (Cain and Oakhill 2011). Therefore, the key to reading at a high level of proficiency is automatic, easy decoding (LaBerge and Samuels 1974). To reach this level of proficiency, learners need to undergo a long learning process (Swart et al. 2017). This labor-intensive learning phase is not easy for all students and can be hindered by a number of factors. A school can only meet the expectations of public education if it can develop the students’ skills and abilities with scientifically based tools. Computer-assisted development programs offer a new way to solve this problem. They can be used to develop pupils’ skills in a targeted way. Thus, a complex, multicomponent development program adapted to the curriculum can be used to facilitate the development of students’ reading skills in the beginning stages of elementary school, one of the methods of which can be technology-based development.

### 1.2. Computer-Based Development of Reading Skills

Computer-assisted education in schools has been around since the 1980s and was identified by Barley et al. (2002) as an effective tool for improving performance among at-risk students. Numerous studies have demonstrated the benefits and success of technology-based development programs (Sivin-Kachala and Bialo 2000; Barley et al. 2002; van Scoter and Boss 2004; James 2014). First, learning in a playful digital environment can enhance motivation, which can lead to increased acceptance, concentration, and persistence in learning tasks (Malouf 1988; Papastergiou 2009). Furthermore, technology-based instruction can reduce cognitive load and contribute to greater retention of course material (Williams and Zahed 1996; Mayer and Moreno 2003; Ricci et al. 2009). Computer-based programs provide opportunities for differentiated instruction of students by enabling real-time data generation and immediate visualization of a student’s performance, which plays a key role in differentiation when a student is deficient, underdeveloped, and in need of additional support (Jenkins et al. 2017; Campbell et al. 2022). With no time limit, all students can progress at their own pace, which can promote their individual development (Corbett 2001). Finally, it can provide personalized, adaptive tutoring without the involvement of instructors or only to a limited extent, which is really beneficial if there are not enough human resources available (Andreev et al. 2009; Athanaselis et al. 2014).

A growing number of computer programs have been developed to promote students’ reading performance (Carlson and Francis 2002; Guthrie et al. 2004; Jamshidifarsani et al. 2019). Most studies deal with the development of one dominant segment of reading, and, most often, developmental procedures prepared for students with some special learning problem (e.g., dyslexia and attention deficit disorder) are evaluated. Most of the procedures support the decoding skills of five- to eight-year-old children in a playful way, which increase students’ motivation. Existing research results have demonstrated the benefits of computer-based development (1) in the development of phonological awareness (Mitchell and Fox 2001; Lonigan et al. 2003; Cassady and Smith 2004; Segers and Verhoeven 2005; Macaruso and Walker 2008; Wild 2009; de Graaff et al. 2009; Nelson 2010; Adlof et al. 2010; Al Otaiba et al. 2012; Savage et al. 2013); (2) in the identification of letter–sound relationships (Segers and Verhoeven 2005; Macaruso et al. 2006); (3) in the area of word recognition skills (van Daal and Reitsma 2000; Hecht and Close 2002; Shelley-Tremblay and Eyer 2009; Macaruso and Rodman 2011; Saine et al. 2011); and in the area of word reading (Johnson et al. 2010; Saine et al. 2011). However, fewer studies deal with curriculum-based complex reading intervention programs and their scientifically proven effects among lower grades without students with special learning needs. The purpose of these development programs is not only to teach decoding skills but also to develop text comprehension.

Kloos et al. (2019) used a similar procedure involving an online reading program called MindPlay Virtual Reading Coach (MVRC) to develop reading in second- and fourth-grade students. The program covers phonological awareness, phonetic skills, vocabulary, grammar, fluency in quiet reading, and comprehension. Fluency in reading was assessed before and after the procedure. MVRC clearly shows an advantage in the area of thin reading. Taken together, the results suggested that increasing the amount of time spent with MVRC directly leads to improved reading fluency. In addition, the program has helped to improve the reading skills of children from middle-class homes, even when reading failure is not directly threatened.

Prescott et al. (2018) used the Core5 online program to improve reading for disadvantaged students. The program component provides a systematic and personalized way to teach reading. The content of the program targets six branches of reading: phonological awareness, phonetics, structural analysis, automation or fluency, vocabulary, and comprehension, which are systematically aligned with the kindergarten standards required to read informative texts and read literature up to fifth grade. Their results in all grades (kindergarten–Grade 5) demonstrated the effectiveness of the program, especially in the early stages of learning reading. Their regression analyses showed that students who made greater progress in the online component scored higher on the reading test. Macaruso et al. (2019) also used the Core5 program to a longitudinal (3-year) study of disadvantaged students. Students began the program in kindergarten and followed their reading scores until the end of the second grade. Their results confirmed the effectiveness of the online development program. First and second graders using the developmental program showed significantly greater reading improvement on a standard test than members of the control group.

Based on a meta-analysis of thirty-two technology-based or technology-assisted reading development programs, Jamshidifarsani et al. (2019) concluded that letter recognition automation can be taught initially, then word recognition automation can be practiced, and, later, phrases, paragraphs, and longer texts can be interpreted. In addition, it was suggested that developers should take advantage of the latest advances in information and communication technologies and design innovative methods that are not available under normal educational conditions.

In summary, the majority of technology-supported development programs to promote reading skills are designed for students with specific learning disabilities and deal with the development of one segment of reading skills. The number of complexes, curriculum-based development programs, which would have been prepared for students who have average abilities and no learning disability but who are struggling with developmental delays for some reason (e.g., online education) is negligible. No such program is known in Hungary at all. Our online reading skills development program was prepared to fill this gap and to speed up students’ development, with the application of which we wanted to eliminate backlogs caused by school closures.

### 1.3. Aims and Research Questions 

This study had two objectives: first, to develop a game-based, personalized reading skills intervention program for third- and fourth-grade students to improve their reading comprehension and close the learning gap in basic reading skills during the first two years of distance education; and, second, to conduct a quasiexperimental research project to test the effect size of the intervention immediately afterward and then three months later in a follow-up test on different groups of students. That is, in the present study, we used a quasiexperimental procedure with propensity score matching to determine the impact of the development program by evaluating students’ comprehension scores. The study addresses the following research issues:

RQ1. How effectively can a complex online reading intervention program be implemented at the ages of 9–11?
RQ2. Which starting level of reading skills is the most sensitive to the complex online training program? Which level can we thus expect the largest effect on?
RQ3. Which group of students can be enhanced the most via the online reading program based on students’ socioeconomic background?
RQ4. How generalizable are the results? Are the effects confirmed by latent-level analyses using a no-change model in the control group and a latent change model in the intervention group?

## 2. Materials and Methods

### 2.1. Participants

The study involved third- and fourth-grade students from 33 schools and 54 classes, for a total of 278 people. To minimize the effect of teachers’ personality and teaching methods, full classes have been involved in the study. Based on students’ pretest performance, we formed learning pairs at the class level, in which one member participated in the development and the other did not. The primary aspect in the formation of the study pairs was that they should be in the same class, as it is guaranteed that the students will master the curriculum with the same methodological repertoire and that their skills will be developed with the same methodology. If more than one student in the same class achieved the same performance, the time spent on the test was also considered a variable. Inclusion of the time factor provided an opportunity to observe the factor of the learner providing an answer immediately or after thinking. 

During data processing, students (1) who lacked a pre-, post-, or follow-up test, (2) who did not participate in 70% of the training (intervention group), or (3) whose time spent on the test did not exceed the minimum time needed to read and complete the tasks were deleted to validate the effectiveness of the program. After data cleaning, propensity score matching was applied: each student in the intervention group was paired with a peer in the control group based on their same school group (classmate) and their performance before the test. In total, 276 participants remained in the research, i.e., 138 pairs of students, indicating a higher number of boys in terms of gender (see Table 1).

### 2.2. Instrument

To evaluate students’ performance, we used a pre-, post-, and follow-up test to frame the online training program, which was implemented on the eDia platform (Csapó and Molnár 2019). The test examined students’ reading comprehension. The pre-, post-, and follow-up tests included the same tasks to measure information search, interpretations, and reflections. Based on the student activity, the online test contains single- and multiple-choice tasks, a total of 28 items. Therefore, the maximum score available on the test was 28 points. Tasks were click/tap or drag & drop. The reliability of the reading comprehension test proved to be good (Npretest = 2700; Cronbach’s αpretest = .859). 

#### 2.2.1. Content and Structure of the Training Program

The online program was primarily designed for third- and fourth-grade students to bridge the learning gap that had arisen during remote learning in reading. Its content was developed in accordance with the national curriculum and the reading books, grammar-spelling textbooks, and other textbooks based on it. Therefore, it can be used for both in-class native language education and individual/group extracurricular catch-up activities.

Its primary function is (1) to develop continuous reading (fluency), (2) to help students understand the text they are reading (comprehension), and (3) to practice grammatical knowledge. The secondary function is to alleviate the socioeconomic and sociocultural disadvantages present in the learning community.

The content of the development program was compiled based on the recommendation of the National Reading Panel (2000), according to which a stronger intervention program includes tasks aimed at developing phonological awareness, sound, text comprehension, and fluency. That is, development of decoding and comprehension skills was realized with varied, multicomponent tasks. With this multicomponent reading intervention, the weaknesses and strengths of each individual can be assessed, and more personalized instruction can be provided.

The texts to be processed are based on texts in the second- and third-grade textbooks. The tasks tied to them are related to the (1) phonological, (2) lexical, (3) syntactic, and (4) semantic linguistic levels. Due to the complexity of the tasks, the development of the morphological-level language is integrated into the language levels (1–4) listed above. Since the students involved in the development have been studying for long periods of time without in-person education in the past two academic years, we considered it necessary to integrate the contents of the second-grade curriculum into the program to eliminate possible lags. The third-grade course starts with the repetition of the second-grade material, the process is simpler, and stress is placed on texts. Therefore, since the development program is embedded in the course, it also fits this line of thinking. In addition, tasks adapted to lower skill levels make up only a small part of the development program. Thus, students who struggle with difficulties have the opportunity to compensate for the gaps, while those who do not struggle with falling behind experience these items as an easy tuning-in task.

The training program contains 15 different texts, designating 15 different development opportunities. On average, each set of tasks contains 13–16 tasks, so the total development program contains 200 basic tasks with additional support instructions and branches. The branching structure of the program allows for tackling the task again with helpful information in the case of an incorrect solution to a task. A task can be completed at 2–3 levels of difficulty. That is, after an unsuccessful answer, the learner is provided with help, and if this is still not enough to reach the correct solution, they can receive further support information. 

The program is tailored to the individual needs of the student, so it includes summaries, explanations, and highlights, which can be listened to, watched, and/or read by the learner, if necessary. Immediate feedback is provided for the children after each task is completed. It takes 20–40 min to complete a series of tasks. The time spent on the task depends to a large extent on the number of support functions that the student is able to use to do the basic task. The instructions, explanations, and additions to the tasks are supplemented by a correctly articulated and emphasized audio file so that students who may have difficulties with reading comprehension can more easily understand the instructions provided. Upon completion of the assignment, the student will receive a summary assessment of their performance. The system and the linear linking of the series of tasks offer an opportunity to interrupt work on the task sequence, and the next time the student enters, they can continue working from where they left off. With this method, the teacher does not have to keep track of which task the student is on, as they can always continue working from the current point, thus avoiding jumping between tasks, and guaranteeing that the student is progressing gradually from the beginning of the task.

**Figure 1:**
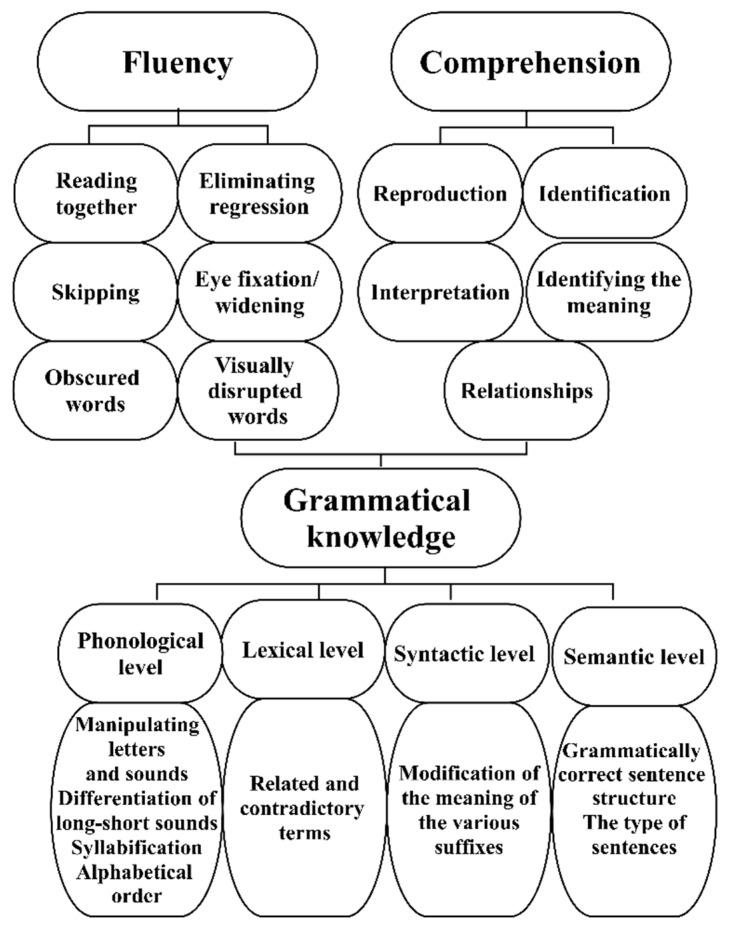
The complex structure of the development program (own editing).

We used a complex content structure to develop reading fluency, comprehension, and correct grammatical structures (Figure 1).

Helping to develop reading fluency is grouped around six types of tasks. Considering the age characteristics, we practiced reading together, where the students first followed the text read out, with an increase in tempo, and then the task was to find the word changed in several places in the text. To eliminate regression, students followed the text read at a normal pace. When skipping, the children looked for accents, punctuation marks, and words in the text. To broaden the eye fixation band, students were only asked to follow words read from the beginning and end of the line, where they were expected to see the distance of initially 2–3 and then 4–5 words. To help them comprehend the word, we practiced reading words spaced out in an increasingly wide band. Then, the task was to read texts with different visual disruptions (blanked letters, incomplete words, scribbled text, and blurred letters).

We assisted in reading comprehension on five levels. During the reproduction, students were expected to repeat a fact in the text. Then, at the level of identification, facts and data were identified. At the third level, the aim of the tasks was to identify the answers that were implicit in the text during production and interpretation. When identifying the meaning, we expected an interpretation of words, word combinations, sentences, and paragraphs during the solution. In addition, the recognition of relationships and connections in the text (e.g., cause–explanation, means–end, cause–effect, etc.) was practiced.

We combined second-grade grammar practice in the above types of tasks, which was designed with special attention to ensure that all language levels are integrated into the tasks. This is how we practiced manipulating letters and sounds; differentiation of long-short sounds; syllabification; alphabetical order; grammatically correct sentence structure; modification of the meaning of the various suffixes and their spelling; types of sentences; and related and contradictory terms. The complexity of the development program is illustrated in Figure 1.

#### 2.2.2. Procedure

The pretest was administered in September–November 2021. After the study pairs were formed, the three-month development started in the second half of November 2021, which was closed in early March 2022 with the administration of the online post-test. Then, in June 2022, we administered another follow-up test. Students completed all the tests and the training tasks online in the computer room at their own institution. The assisting educators were given detailed written and, if necessary, oral instructions on the purpose of the tasks in the development program and the manner of implementation. The teachers were not allowed to help students during the testing and training process beyond the login procedure.

We used the propensity score matching technique to arrange the students into pairs, minimizing the influence of factors affecting change. In connection with the algorithm, in addition to the skill level of the students, which was characterized by their average performance on the test, we considered the student’s school, class (excluding the various effects resulting from the teacher’s classroom work), gender, and grade (excluding gender and grade differences in development). In summary, the average performance of the students on the test was taken as the primary basis during the propensity score matching technique.

In addition to descriptive statistics, we used a two-sample *t*-test to analyze the differences between disadvantaged and nondisadvantaged students. A paired *t*-test was used to examine the differences in performance between the third and fourth graders between pre- and post-development and then three months later at the sample level. Cohen’s d (Cohen 1988) was used to describe the magnitude of effect size, that is, the changes in standard deviation units. If its value is less than .2, it is considered a small effect; if it is around .5, it is a medium effect size; and if it is greater than .8, it is interpreted as a large effect (Cohen 1988).

Beyond the analyses using observed variables, which have several limitations (Alessandri et al. 2017), we also used latent-curve modeling and a three-step approach (Little et al. 2002) to evaluate the generalizability of the results on a latent level. By comparing the relative fit indices of the models, we gained further insights into students’ development as a result of regular school instruction (control group) and students’ development as a result of explicit training beyond regular school instruction. First, we specified a no-change model for both groups (intervention and control), assuming that neither normal school education nor the additional intervention had produced any meaningful effect. In this model, the mean and variance of the second-order intercept factor were freely estimated across groups. Second, we used a latent change model for the intervention and a no-change model for the control group. That is, we additionally estimated a slope growth factor in the intervention group to capture any possible change. Finally, we estimated a latent change model for both groups. We compared model fit indexes, CFI (Comparative Fit Index) and TLI (Tucker–Lewis Index) with associated 90% confidence intervals, and RMSEA (Root Mean Square Error of Approximation) and the changes in fit indexes between the different models. We accepted CFI and TLI values > .90 and RMSEA values < .08 (see Kline 2016). The Akaike information criterion (AIC; Burnham and Anderson 2004) was also used, as it “rewards goodness of fit and includes a penalty that is an increasing function of the number of parameters estimated” (Alessandri et al. 2017). If the referring fit index of the model differs more than 2 from the best fitting model, it has considerably less support. If the difference is larger than 10, there is no support for that model. The differences between the CFI and RMSEA values were also used in identifying the best fitting model. According to Chen (2007), if differences between CFI and RMSEA values of two different models exceed .01, the data supports the models on a different level. Probabilistic model selection based on information criteria provides an analytical technique for scoring and choosing among candidate models. The Akaike information criterion (AIC) and the Bayesian information criterion (BIC) are used for model selection among a finite set of models. Both are based on likelihood function. Generally, models with lower AIC and BIC are preferred; however, they do not offer information about the absolute quality of a model, only the quality relative to each of the other models. Thus, AIC and BIC also provide tools for model selection beyond the fit indices (CFI, TLI, and RMSEA).

## 3. Results

### 3.1. RQ1. Changes in Reading Performance Compared to Students’ Original Reading Skills Level

In our research, we first examined the comprehension performance of the intervention and control groups before the intervention with the pretest. Students’ performance was treated as a continuous variable. In both groups, the condition of homogeneity of variance was met (F_pretest_ = 1.21, *p* = .27). The difference between the two groups was not significant on the pretest (t = −1.18, *p* = .24). To examine the effectiveness of the development program, we compared the frequency of student performance, based on the three measurement occasions, that is, on the pretest (M_ig_ = 56.25; SD_ig_ = 20.99; M_ig_ = 59.10; SD_cg_ = 19.24), post-test (M_ig_ = 67.58; SD_ig_ = 17.48; M_cg_ = 61.16; SD_cg_ = 20.99), and follow-up test (M_ig_ = 68.57; SD_ig_ = 20.05; M_cg_ = 67.44; SD_cg_ = 18.36). 

**Figure 2:**
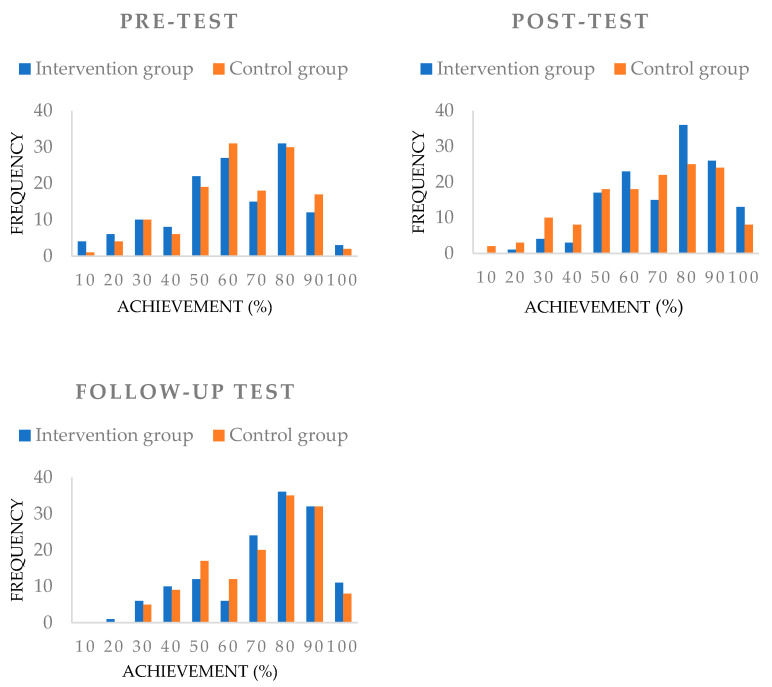
Frequency distribution of intervention and control group performance at three times: (1) pretest: before intervention; (2) post-test: after intervention; and (3) follow-up test: three months after intervention.

Figure 2 illustrates the frequency distribution of the reading comprehension performance of the two student groups measured at three times (pretest, post-test, and follow-up test). The performance of the two groups overlapped well before the start of the study; there were three positive shifts in the performance of the intervention group compared to the control group after the intervention. (1) In the intervention group, there was a decrease in the number of students performing at or below 50 percent after the intervention, and (2) the number of students completing between 60 and 100 percent grew compared to the control group. (3) Three months later, the number of students completing between 50 and 60 percent fell further compared to members of the control group. That is, the intervention group was able to maintain its post-intervention skills advantage over the control group even three months after the intervention.

**Figure 3:**
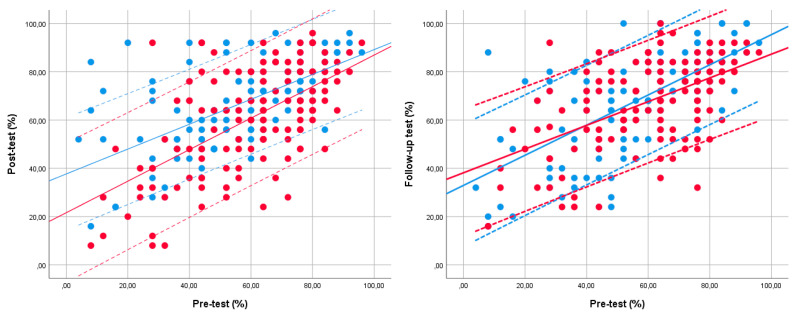
Comparison of student performance on the pre- and post-tests and on the pre- and follow-up tests.

The relationship between students’ performance on the pre- and post-tests and on the pre- and follow-up tests is illustrated in Figure 3. The first figure shows the power of change between the pre- and post-tests, and the second figure demonstrates the power of change between the pre- and follow-up tests. The abscissa indicates the performance on the pretest, while the ordinate represents the performance on the post- or follow-up test. Each dot in the figure symbolizes a student. The blue color stands for the intervention group, and the red color signifies the control group. Students whose symbols fall on the mean line or between the two dashed lines (representing a standard deviation) performed equally in both cases. If the symbol is above the dashed line, it means that the student has shown a significant improvement from the pre- to the post-test or from the pre- to the follow-up test, while if it is below the dashed line, the student performance was significantly worse from the first to the second and from the first to the third data collection.

Based on the results, it can be concluded that most members of both groups performed better on the post-tests than on the pretest. However, it is also observed that this statement is not true for all students, as we find individuals with a weaker performance on the post- or follow-up test. 

The development of the students in the intervention and control groups between pre- and post-tests and pre- and follow-up tests in standard deviation units on a manifest level are shown in Table 2. As a result of the extra development, the children developed by half a standard deviation (d = .51, t = −6.65, *p* < .01). During the same time, there was no development in this area in the control group (d = .03, t = −.43, *p* = .67). Between the post-test and the follow-up test, students participated exclusively in school education, where the previously developed intervention group improved by one-tenth of a standard deviation (d = .12, t = −1.24, *p* = .22), while the control group developed by three-tenths of a standard deviation (d = .35, t = −5.33, *p* < .01). Since the intervention and control groups started from the same level at the beginning of the research, it is likely that this is the effect of accelerated development—where those at higher levels are presumably less developed. Overall, both groups underwent marked development as a result of school education and extra development (d_ig_ = .59, t_ig_ = −7.72, p_ig_ < .01; d_cg_ = .39, t_cg_ = −4.68, p_cg_ < .01); that is, the period involved was sensitive to the development of this skill, while the development of the intervention group proved to be more marked.

### 3.2. RQ2. Expand the Impact of the Intervention According to the Initial Skill Level of the Students

To monitor the effectiveness of the training as regards students’ starting level of reading skills, we divided students into three groups based on their performance on the pretest (Table 3). Students in the first group (N = 42) were labeled low achievers, performing more than one standard deviation lower (0–39%) than the mean achiever in the second group (N = 139, Mean = 54.20%, SD = 8.98; 40–78%). Students in the third group (N = 95), who were called high achievers, managed one standard deviation higher than students in the second group (79–100%).

**Figure 4:**
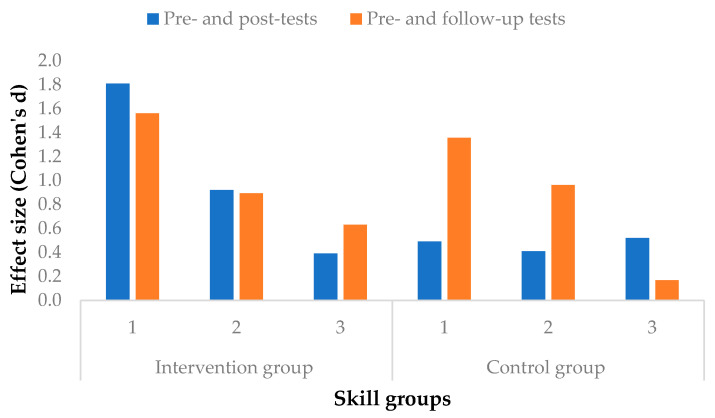
Change in effect size by skill groups in the interventional group and control group. Skill groups (performance on pretest): 1: 0–34%; 2: 35–67%; 3: 68–100%.

The standardized differences between the control and intervention groups proved to be much higher in the two lower-skilled groups in the intervention group than that of the control group (d_ig1_ = 1.81, t_ig1_ = −5.98, p_ig1_ < .01; d_cg1_ = .49, t_cg1_ = −1.60, p_cg1_ = .13; d_ig2_ = .92, t_ig2_ = −6.75, p_ig2_ < .01; d_cg2_ = .41, t_cg2_ = −2.65, p_cg2_ = .01). In the third skill group, the performance of the students in the control group was slightly higher than that of the intervention group (d_ig3_ = .39, t_ig3_ = −2.64, p_ig3_ = .01; d_cg3_ = .52, t_cg3_ = 3.06, p_cg3_ < .01). There was a marked change in the two lower capacity ranges of the intervention group. Development was accelerated in these two groups. After three months, as a result of explicit school development, there was a further slight improvement in all three skill groups in the intervention group, while marked progress was seen in the two lower-skilled groups in the control group (d_cg1_ = .63; d_cg2_ = .39). However, the third skill group in the control group did not show any improvement. That is, the extra development not only sped up the skills of the low- or medium-achieving students but also that of the high-performing students, whose skills showed marked improvement after three months (Figure 4).

### 3.3. RQ3. Expand the Effect Size of the Intervention on Disadvantaged Students

**Figure 5:**
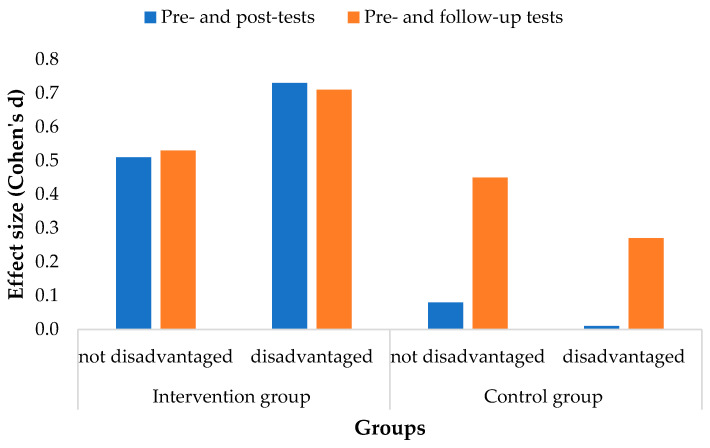
The effect size of the development program in terms of disadvantage in the intervention and control groups.

As one of the priorities of school education is to bridge the gap experienced by socioeconomically disadvantaged students, we were interested in the extent of the developmental impact of the intervention program on them. Disadvantaged students are students who experience normal school conditions, who receive regular family services support because of their parents’ low educational attainment and/or under- or unemployment, and/or whose living or housing conditions are inadequate. The performance of the students in the intervention and control groups according to the three skill groups on the pre-, post-, and follow-up tests with respect to disadvantage is shown in Table 4. In terms of disadvantage, the distribution of students was similar in the intervention and control groups. The performance measured on the pretest was lower in both groups in the intervention group, and the results obtained on the post-test show higher values compared to the same subgroups in the control group. The magnitude of the effect of the experimental intervention is illustrated in Figure 5.

The intervention had a positive effect on the intervention group. The value of the developmental effect was median for the disadvantaged (d = .72) and nondisadvantaged students (d = .51). At the same time, the control group showed no improvement. Three months after the experimental intervention, there was no change in the skill of the intervention group as a result of explicit school development. At the same time, there was a slight improvement in the performance of the control group. This development was lower among the disadvantaged students (d = .27) than among the nondisadvantaged students (d = .45). That is, the intervention greatly accelerated the development of the disadvantaged and nondisadvantaged students, so both groups are sensitive to the development of these skills (Figure 5).

The development of the students in the intervention and control groups between pre- and post-tests and between post- and follow-up tests on a manifest level in standard deviation units by skill group and disadvantaged situation are shown in Table 5. The number of students in the same skill groups was similar in the intervention group and the control group. In the intervention group, the students of the disadvantaged high-skilled group did not develop due to the intervention; however, in the other subgroups, the intervention resulted in a marked improvement. The development of the low-skilled disadvantaged group was most marked (d = 2.04). School development led to a small improvement in the low-skilled disadvantaged students (d = .39), and no progress was made in the two higher-skilled groups in the control group. After the end of the experiment, only the high-skilled students developed in the disadvantaged intervention group (d = .66), and school development did not contribute to the change in the other two skill groups.

### 3.4. RQ4. Evaluating the Effect of the Intervention Program within the Latent Curve Modeling Framework

The reading comprehension test monitored the areas of information search, interpretation, and reflection in 9–11-year-old students. First, we tested a measurement model for comprehension with all three indicators combined under one general factor. The measurement model based on the pretest results showed an acceptable fit (χ^2^ = 496.1; df = 273; *p* < .05; CFI = .926; TLI = .918; RMSEA = .055 (CI: .047, .053)). Second, we created two parallel forms of the comprehension scale based on the factor loading values. This 2-dimensional model showed a better fit (χ^2^ = 421.5; df = 272; *p* < .05; CFI = .950; TLI = .945; RMSEA = .045 (CI: .036, .153)), so in further analyses, we used the latent growth model. Third, to run a latent change model by the analyses, at least two indicators per time point are required. Therefore, based on the factor loading values and the procedure described by Steyer et al. (1997) and Little et al. (2002), we created two parcels for each time point both on the test and dimension levels. The composition of the parcels was identical for each of the three time points. Table 6 shows the fit indexes for the three alternative models. According to the fit indexes, the third model fitted the data the best (CFI = .842; TLI = .828; RMSEA = .255 (CI: .212, .299)). Information criteria also supported these results (determined by the FIT index).

In order to test the developmental effect on a latent level, we analyzed the development between T1 (development between pre- and post-tests) and T2 (development between pre- and follow-up tests) time on the level of dimensions for the intervention and control groups separately applying Alessandri et al.’s (2017) description of evaluation intervention programs with a pretest–post-test design. Table 7 shows the fit indexes for the three alternative models in the first dimension (development between pre- and post-test). According to the fit indices, even in the first dimension, the third model fitted the data the best (CFI = .930; TLI = .923; RMSEA = .129 (CI: .084, .177)). Information criteria also supported these results (determined by the FIT index).

Table 8 shows the fit indexes for the three alternative models in the second dimension (development between the pre- and follow-up tests). According to the fit indices, even in the second dimension, the third model fitted the data the best (CFI = .925; TLI = .924; RMSEA = .289 (CI: .249, .331)). Information criteria also supported these results (determined by the FIT index).

During the SEM analyses, we tested whether the three areas (information search, interpretation, and reflection) should be treated and interpreted as separate dimensions within reading comprehension skills or whether the use of a one-dimensional construct is recommended based on the data, i.e., whether it is sufficient to include performance on the test in the analyses. In the latter case, all the items on the test were classified as manifest variables into a common dimension, and the latent variable of reading text comprehension was constructed. In the case of the multidimensional model, we built the individual dimensions as latent variables from the items on the individual subtests as manifest variables. Based on the results, it can be concluded that, in all cases—on a construct and a dimension level—the third model fitted the data the best; that is, the effect of the development was also confirmed on a latent level. 

## 4. Discussion

The study presents an online reading skills development program focused on developing comprehension and follows a quasiexperimental design with a total of 276 third and fourth graders. We used a quasiexperimental procedure with propensity score matching to determine the impact of the development program by evaluating students’ comprehension scores. The goal was to eliminate the learning gap in reading skills accumulated during distance learning for students aged 9–11 using a curriculum-based, playful reading skills development program and testing the effect of the quasiexperimental project immediately after the intervention and three months later for different groups of students.

### 4.1. RQ1. Changes in Reading Performance Compared to Students’ Original Reading Skills Level

In our first question, we examined changes in performance relative to students’ original reading skill levels. Previous research has already examined the usability and effectiveness of technology-based education at the school level (including Sivin-Kachala and Bialo 2000; Barley et al. 2002; van Scoter and Boss 2004; James 2014) and the potential for developing technology-based comprehension in addition to normal school teaching (e.g., Jenkins et al. 2017; Kloos et al. 2019; Campbell et al. 2022). Their results bore out the success of technology-based reading development. Our research results confirmed that the application of the training program in the sample is suitable for improving comprehension performance. We found that the text comprehension of the students involved in the development improved by half a standard deviation (d = .51) after the completion of the development program, while there was no change in the skill level of the control group members (d = .03). In the three months between the pretest and the follow-up test, students only received school education, where we experienced a positive change in the skill levels of both groups. That is, this period is sensitive to the development of comprehension. Since students started from the same skill level before beginning the experiment, we can conclude that extracurricular development accelerated the development of the intervention group, as the students involved in the development retained their marked development.

### 4.2. RQ2. Expanding the Impact of the Intervention According to the Initial Skill Level of the Students

As regards the second research question, which aimed to gain more knowledge about the efficacy of the intervention program, we examined its effect size according to students’ level of skill. Based on the results, we concluded that the intervention program was able to speed up the development of the students in the intervention group and that students in both lower skill groups were most affected by the training. The worst performing students (Skill Group 1) showed the greatest improvement, with the rate of impact of the intervention being large (d = 1.81) and the moderately performing students (Skill Group 2) being medium (d = .92). Strongly performing students (Skill Group 3) showed the least improvement (d = .23). Overall, after the completion of the training program, there was a positive change in comprehension among the members of all three skill groups compared to the members of the control group; that is, their development accelerated. As a result of the measurement three months later, it can be concluded that their performance advantage was maintained by the lower- and higher-skilled intervention groups; however, this advantage decreased. Those with good skills were able to maintain their marked advantage, with their comprehension improving by more than two-tenths of a standard deviation (d = .24), and the students in the first skill group improved by an additional one-tenth of a standard deviation (d = .12). Our results are partially consistent with Campbell et al. (2022), who also found that their development was effective for students at the highest and lowest levels of the study. We consider these results to be particularly important, as students were lacking in-person schooling for two school years, resulting in a significant learning gap (Engzell et al. 2021; Tomasik et al. 2021; Molnár and Hermann 2022). The positive changes in the performance of the intervention group suggest that the development program is also suitable for overcoming these disadvantages.

### 4.3. RQ3. Expanding the Effect Size of the Intervention on Disadvantaged Students

In our third research question, we examined the extent of the impact of the intervention on disadvantaged students. In a three-year longitudinal study, Macaruso et al. (2019) found that disadvantaged students experienced a slippage in reading performance each summer, performance that was successfully overcome by students in development each year by supplementing Core5 lessons. Our results show that the text comprehension of the disadvantaged students involved in the development program improved by half a standard deviation (d = .53) after the completion of the program, while the control group developed by three-tenths of a standard deviation (d = .32). Based on our results three months later, we can conclude that due to accelerated development, the comprehension of at-risk students in development improved by one-sixth of a standard deviation (d = .66), while there was no change in explicit school development in the lower-skilled groups. These results are consistent with other findings, showing that effective interventions may be beneficial for at-risk learners (Connor et al. 2013; Lovett et al. 2017; Simmons et al. 2008; Macaruso et al. 2019), especially at the beginning of the school year, to make up for the summer slippage.

### 4.4. RQ4. Evaluating the Effect of the Intervention Program within the Latent Curve Modeling Framework

Our fourth question involved an evaluation of the impact of the intervention program in the latent curve modeling framework. The developmental power of the intervention program was confirmed by structural equation modeling analyses. Three different combinations of the no-change and latent change models were used in both the intervention and control groups. The best-fit trajectory (latent change model) and the significant positive latent slope factor of the intervention group confirmed the result obtained at the manifest level as regards the positive effect of training in both dimensions, while the students in the control group showed no significant change at the latent level. Importantly, our results also demonstrated that there were significant differences between students in their response to the training program, as indicated by the interaction between treatment and baseline.

In summary, the results indicate that the development of this online training program can be considered a success. It develops third to fourth graders in a playful environment. The findings suggest that reading skills can develop significantly and effectively not only traditionally in person, but also in a computer environment. The development program has achieved its goal because it truly focuses on catching up lower-skilled and/or disadvantaged groups. Surprisingly, however, it also significantly facilitated the development of students in the higher-skilled group. Therefore, this development program can be used at the classroom level as a complement to school learning to accelerate the development of comprehension.

## 5. Limitations of the Study

The limitations in the study affected the sample and methodological sections. It used convenience sampling, as schools and classes were able to join the sample on a voluntary basis, so representativeness did not appear. The students who completed the pretest dropped out significantly during the development process, an exploration of which requires further research. Although the pairs of learners were fitted according to certain criteria, no background variables were considered, nor was the effect of the reading teaching method on development.

## 6. Conclusions

The study presents a reading skills development program for third to fourth graders using a quasiexperimental design. Based on our research results, we can conclude that our complex program designed to improve reading works effectively. The online development program accelerated development and aided students involved in the program in gaining a significant developmental advantage over their control group peers. The results of the program also showed that the development of comprehension can also take place in an online environment, which offers an objective form of measurement and development for teachers and students. The uniqueness of our program lies primarily in the fact that its content has been developed in line with the national curriculum and recommended textbooks used in Hungary and can therefore be used in class and extracurricular activities. Second, it adapts to the needs and abilities of the students because its branching structure guides students to the right solution with helpful information, explanations, and highlights. It is therefore also suited to differentiated learning. Thirdly, it is simple to use and does not require the presence of a specialist to implement development. The use of the online development program is not tied to a strict time. It can be started at any time of the school year and day.

## Figures and Tables

**Table 1:** Gender distribution of the sample.

	Gender (%)		
	Boys	Girls	Missing
Intervention group	49.3	44.9	5.8
Control group	60.1	34.8	5.1
Total	54.7	39.9	5.4

**Table 2:** The development of the students in the intervention and control groups between the pre-, post-, and follow-up tests in standard deviation units on a manifest level.

Group	Test	Development	SD	t	df	*p*	d
Intervention group	Pre- and post-tests	9.68	17.11	−6.65	137	<.01	.51
	Post- and follow-up tests	1.58	14.85	−1.24	136	.22	.12
	Pre- and follow-up tests	11.04	16.72	−7.72	136	<.01	.59
Control group	Pre- and post-tests	.67	18.09	−.43	137	.67	.03
	Post- and follow-up tests	6.69	14.68	−5.33	136	<.01	.35
	Pre- and follow-up tests	7.27	18.18	−4.68	136	<.01	.39

**Table 3:** Performance of students in the intervention and control groups according to the three skill groups on the pre-, post-, and follow-up tests.

Skills Group	Test	Group	N	M	SD	t	*p*
1	Pretest	Intervention group	23	22.26	10.43	−.95	.35
		Control group	19	25.05	8.09		
	Post-test	Intervention group	23	49.57	20.95	2.74	<.01
		Control group	19	32.00	20.31		
	Follow-up test	Intervention group	23	48.52	22.25	.46	.65
		Control group	19	45.53	19.51		
2	Pretest	Intervention group	69	54.84	9.85	−.90	.37
		Control group	70	56.34	9.74		
	Post-test	Intervention group	69	64.64	13.78	1.47	.14
		Control group	70	60.63	17.99		
	Follow-up test	Intervention group	69	66.43	17.17	−.54	.59
		Control group	70	67.94	15.55		
3	Pretest	Intervention group	46	79.74	6.39	−.08	.94
		Control group	49	79.84	5.77		
	Post-test	Intervention group	46	80.43	9.17	3.22	<.01
		Control group	49	72.90	13.15		
	Follow-up test	intervention group	46	82.96	9.92	2.82	<.01
		Control group	49	76.24	12.95		

**Table 4:** The performance of the students in the intervention and control groups according to the three skill groups on the pre-, post-, and follow-up tests with respect to disadvantage.

Disadvantaged Situation	Test	Groups	N	Mean	SD	t	*p*
Nondisadvantaged	Pretest	Intervention group	104	62.38	18.47	−.22	.82
		Control group	109	62.94	17.74		
	Post-test	Intervention group	104	70.35	16.82	2.33	.02
		Control group	109	64.55	19.27		
	Follow-up test	Intervention group	104	72.27	18.93	.61	.55
		Control group	109	70.79	16.73		
Disadvantaged	Pretest	Intervention group	34	43.41	23.53	−1.24	.22
		Control group	29	50.76	23.42		
	Post-test	Intervention group	34	58.35	16.69	2.11	.04
		Control group	29	47.86	22.74		
	Follow-up test	Intervention group	34	58.82	19.76	.46	.65
		Control group	29	56.59	18.57		

**Table 5:** Development of students in the intervention and control groups between pre- and post-tests and between post- and follow-up tests on a manifest level in standard deviation units by skill group and disadvantaged situation.

Group	Disadvantaged Situation	Skill Group	Tests	N	Development	SD	t	*p*	d
Intervention group	Nondisadvantaged	1	Pre- and post-tests	9	25.51	27.24	−2.81	.02	1.42
			Post- and follow-up tests		2.92	23.24	.38	.72	0
		2	Pre- and post-tests	55	11.25	14.31	−5.83	.00	.97
			Post- and follow-up tests		1.67	14.24	−.87	.39	.10
		3	Pre- and post-tests	40	3.61	8.15	−2.80	.01	.45
			Post- and follow-up tests		1.99	10.33	−1.22	.23	.16
	Disadvantaged	1	Pre- and post-tests	14	31.48	21.29	−5.53	.00	2.04
			Post- and follow-up tests		−2.23	22.82	.37	.72	.00
		2	Pre- and post-tests	14	9.52	9.38	−3.80	.00	.82
			Post- and follow-up tests		.04	13.66	.01	.99	.00
		3	Pre- and post-tests	6	.62	4.33	.35	.74	.00
			Post- and follow-up tests		3.86	6.32	−1.50	.20	.66
Control group	Nondisadvantaged	1	Pre- and post-tests	9	9.88	24.91	−1.19	.27	.58
			Post- and follow-up tests		14.98	12.30	−3.65	.01	.62
		2	Pre- and post-tests	58	7.47	18.24	−3.12	.00	.53
			Post- and follow-up tests		6.21	15.55	−3.04	.00	.37
		3	Pre- and post-tests	42	−5.03	12.16	2.68	.01	.00
			Post- and follow-up tests		4.60	11.18	−2.67	.01	.37
	Disadvantaged	1	Pre- and post-tests	10	5.56	17.22	−1.02	.33	.39
			Post- and follow-up tests		10.05	12.03	−2.64	.03	.69
		2	Pre- and post-tests	12	2.78	14.91	.65	.53	.00
			Post- and follow-up tests		8.22	11.27	−2.53	.03	.55
		3	Pre- and post-tests	7	−6.88	12.55	1.45	.20	.00
			Post- and follow-up tests		−3.00	21.30	.37	.72	.00

**Table 6:** Goodness-of-fit indices for the tested models on test level—between T1 and T2.

Model	χ^2^	df	AIC	BIC	CFI	TLI	RMSEA [90% CI]
Latent change model for both of the groups	103.2	10	8837.8	8901.9	.842	.810	.268 [.222, .316]
No-change model for both of the groups	155.4	12	8885.9	8942.9	.757	.757	.303 [.262, .347]
No-change model for the control and latent change model for the intervention group	108.6	11	9331.7	9393.2	.842	.828	.255 [.212, .299]

Note: CFI = Comparative Fit Index; TLI = Tucker–Lewis Index; RMSEA = Root Mean Square Error of Approximation; CI = confidence interval; AIC = Akaike information criterion; BIC = Bayesian information criterion.

**Table 7:** Goodness-of-fit indices for the models tested on the test level in the first dimension (development between the pre- and post-tests).

Model	χ^2^	df	AIC	BIC	CFI	TLI	RMSEA [90% CI]
Latent change model for both groups	35.8	10	9902.7	9967.8	.928	.913	.137 [.091, .187]
No-change model for both groups	54.9	12	9917.8	9975.6	.880	.880	.162 [.120, .206]
No-change model for the control group and latent change model for the intervention group	36.1	11	9900.9	9962.4	.930	.923	.129 [.084, .177]

Note: CFI = Comparative Fit Index; TLI = Tucker–Lewis Index; RMSEA = Root Mean Square Error of Approximation; CI = confidence interval; AIC = Akaike information criterion; BIC = Bayesian information criterion.

**Table 8:** Goodness-of-fit indices for the models tested on the test level in the second dimension (development between the pre- and follow-up tests).

Model	χ^2^	df	AIC	BIC	CFI	TLI	RMSEA [90% CI]
Latent change model for both groups	144.0	10	8671.9	8737.0	.925	.911	.313 [.269, .359]
No-change model for both groups	149.2	12	8671.8	8733.2	.924	.918	.299 [.257, .343]
No-change model for the control group and latent change model for the intervention group	145.8	11	8671.2	8731.0	.925	.924	.289 [.249, .331]

Note: CFI = Comparative Fit Index; TLI = Tucker–Lewis Index; RMSEA = Root Mean Square Error of Approximation; CI = confidence interval; AIC = Akaike information criterion; BIC = Bayesian information criterion.

## Data Availability

Data are available upon request due to privacy restrictions.
